# Serological diagnosis of canine leishmaniosis: comparison of three commercial ELISA tests (Leiscan^®^, ID Screen^®^ and *Leishmania* 96^®^), a rapid test (Speed Leish K^®^) and an in-house IFAT

**DOI:** 10.1186/1756-3305-7-111

**Published:** 2014-03-24

**Authors:** Laia Solano-Gallego, Sergio Villanueva-Saz, Marta Carbonell, Michele Trotta, Tommaso Furlanello, Alda Natale

**Affiliations:** 1 Departament de Medicina i Cirurgia Animals, Facultat Veterinaria, Universitat Autònoma de Barcelona (UAB), Barcelona 08193, Cerdanyola, Spain; 2 Laboratorio Veterinario San Marco, Padova, Italy; 3 Istituto Zooprofilattico Sperimentale delle Venezie, Padova, Italy

**Keywords:** *Leishmania infantum*, Dog, ELISA, IFAT, Serological rapid test and vaccine

## Abstract

**Background:**

Speed Leish K^®^ is used as a serological screening test for *Leishmania* infection prior to vaccination. Limited comparative serological studies with Speed Leish K^®^ have been performed. The aim of this study was to evaluate the diagnostic performance of four commercially available serologic tests including ELISAs (Leiscan^®^, ID Screen^®^ and *Leishmania* 96^®^), a rapid test (Speed Leish K^®^) and an in-house IFAT for the detection of specific antibodies against *Leishmania infantum* antigen in dogs in different states of infection.

**Methods:**

Sick infected dogs (n = 36), healthy infected dogs (n = 18), *L. infantum* seropositive dogs with low to high levels of antibodies (n = 53), dogs seropositive to other pathogens (to evaluate cross reaction) (n = 14) and uninfected dogs from a non-endemic area (n = 50) and from an endemic area (n = 32) were analysed by the serological methods mentioned above.

**Results:**

The sensitivity was as follows: ID Screen^®^ (0.953), Leiscan^®^ and *Leishmania* 96^®^ (0.925), IFAT (0.869) and Speed Leish K^®^ (0.636). The maximum specificity (1.000) was attained for all diagnostic tests except the *Leishmania* 96^®^ (0.896) and IFAT (0.917). The accuracy was as follows: ID Screen^®^ (0.975), Leiscan^®^ (0.961), *Leishmania* 96^®^ (0.911), IFAT (0.892) and Speed Leish K^®^ (0.808). In relation to the area under the ROC curve (AUC-ROC), the maximum value was attained with the ID Screen^®^ (0.993) closely followed by Leiscan^®^ (0.990), then, *Leishmania* 96^®^ (0.962), IFAT (0.926) and Speed Leish K^®^ (0.818). For the Kappa index, the best result was obtained by the ID Screen^®^ (0.951) followed by Leiscan^®^ (0.921), *Leishmania* 96^®^ (0.822), IFAT (0.783) and Speed Leish K^®^ (0.622). Statistically significant differences were found between the AUC-ROC of quantitative serological tests and the only qualitative rapid test evaluated. There were also statistically significant differences between AUC-ROC of the ELISAs (ID Screen^®^ and Leiscan^®^) and IFAT.

**Conclusions:**

Leiscan^®^ and ID Screen^®^ had superior diagnostic performance measures than IFAT and all quantitative serological tests were superior when compared to Speed Leish K^®^. Thus, Speed Leish K^®^ may be considered a less valuable screening test prior to vaccination as it may result in vaccination of seropositive dogs and in some cases seropositive sick dogs.

## Background

Canine leishmaniosis (CanL) is a vector-borne zoonotic disease caused by *Leishmania infantum,* endemic in more than 70 countries in the world. It is present in regions of southern Europe, Africa, Asia, South and Central America [1]. Dogs are the main reservoir for this infection and sandflies are the only arthropods that are adapted to its biologic transmission. However, other non-sandfly proven ways of infection include blood transfusion, vertical and venereal transmission [2,3].

In endemic areas, the prevalence of *L. infantum* infection in dogs is greater than the seroprevalence and the prevalence of clinical disease [1,4]. Therefore, CanL is a good example of a disease in which infection does not equal clinical illness due to the high prevalence of persistent subclinical infection. In addition, clinical illness varies from self-limiting disease to very severe fatal disease. Clinical staging of CanL includes four stages of severity of illness based on clinical signs, clinicopathological abnormalities and serology. For these reasons, diagnosis of this parasitic infection and its clinical manifestations can be complex [2,3].

The biggest obstacle in the evaluation of diagnostic tests for CanL is that there is not a definitive diagnostic reference test or gold standard with which to compare the alternative diagnostic assays. There is no diagnostic test with 100% sensitivity and 100% specificity for detection of *L. infantum* infection and therefore it is essential to know the terms and limitations of each diagnostic test, and to select the best tests for the purpose of the diagnosis [3].

The methods used for diagnosis of dogs with suspected clinical leishmaniosis include the detection of amastigotes in stained cytological smears of aspirates or histopathological sections from several tissues. Immunohistochemical staining of tissue sections is employed to enhance the visualization of the parasite. The isolation in culture of parasites from infected tissues is not suitable for rapid diagnosis. However, the most useful diagnostic approaches for investigation of infection in sick and healthy sub-clinically infected dogs include: (1) detection of specific serum anti-leishmanial antibodies by quantitative serological techniques and (2) demonstration of the parasite DNA in tissues by applying molecular techniques. High antibody levels are usually associated with disease and a high parasite density and, for this reason, they are conclusive of a diagnosis of leishmaniosis. However, the presence of lower antibody levels is not necessarily indicative of patent disease and needs to be confirmed by other diagnostic methods such as polymerase chain reaction (PCR), cytology or histology [2,3].

As mentioned above, serological methods are the most common diagnostic techniques used for the diagnosis of CanL. A vaccine, CaniLeish^®^ (Virbac, France), has recently been licensed in Europe for the prevention of CanL in seronegative dogs. The manufacturers recommend the use of a rapid serological test, Speed Leish K^®^, prior to vaccination as a screening test for *Leishmania* infection [5]. However, so far, only one comparative serological study with this rapid test has been published and therefore, the information about the diagnostic performance of this assay is extremely limited [6]. Moreover, there are several different commercial serologic tests currently available, however their effectiveness might vary widely and therefore affects the ability to reach a correct diagnosis.

For these reasons, the aim of this study was to evaluate the diagnostic performance of four commercially available serologic assays including three quantitative commercial enzyme-linked immunosorbent assay (ELISA) tests (Leiscan^®^, ID Screen^®^ and *Leishmania* 96^®^), one qualitative commercial rapid test (Speed Leish K^®^) and one quantitative in-house indirect fluorescent antibody test (IFAT) for the detection of specific antibodies against the antigen of *L. infantum* in dogs with different states of infection. In the present manuscript, the diagnostic performance of quantitative and qualitative serological tests is reported.

## Methods

### Serological techniques

#### Commercial tests

Three commercial ELISAs and one immunochromatographic test were evaluated. The ELISA based quantitative assays were: the Leiscan^®^*Leishmania* ELISA Test (Esteve Veterinaria, Laboratorios Dr. Esteve SA, Spain), ID Screen^®^ Leishmaniasis Indirect Test (VET-Innovate ID Diagnostics, France) and *Leishmania* 96^®^ (Agrolabo S.p.A., Italy). The immunochromatographic based qualitative assay was: Speed Leish K^®^ (Virbac, France). Assays were carried out according to the manufacturer’s instructions. In the commercial ELISA tests, all samples were analyzed in duplicate.

#### In house techniques

The in house IFAT was performed at the *Istituto Zooprofilattico Sperimentale delle Venezie* (Padova, Italy) and is described in the Manual of the World Organisation for Animal Health [7]. The antigen was prepared from promastigotes of *L. infantum* from *Istituto Superiore di Sanità* (Italy). Anti-*Leishmania* antibodies were detected using anti-dog IgG conjugated to fluorescein isothiocyanate (Sigma-Aldrich, USA). Samples were classified as positive if promastigote cytoplasmatic or membrane fluorescence was observed at a serum dilution of 1:40 or higher.

The *Universitat Autònoma de Barcelona* (UAB) in house ELISA was performed on sera of all dogs studied as the reference quantitative serological technique as previously described [4,8-10], with some modifications. This UAB in house ELISA has good diagnostic performance [4,8-10]. Briefly, dog sera were diluted to 1:800 and incubated in sonicated crude *L. infantum* antigen-coated plates (20 μg/mL) for 1 hour at 37°C. The plates were then washed with 0.05% Tween 20 in phosphate-buffered saline (PBS) and incubated with Protein A conjugated to horseradish peroxidase (1:30,000 dilution; Sigma-Aldrich) for 1 hour at 37°C. Plates were washed again with 0.05% PBS-Tween 20. The plates were developed by adding the substrate solution *ortho*-phenylene-diamine and stable peroxide substrate buffer (Thermo scientific). The reaction was stopped with 50 μl of 2.5 M H_2_SO_4_. Absorbance values were read at 492 nm in an automatic microELISA reader (ELISA Reader Anthos 2001). All plates included the serum from a sick dog with a confirmed infection as positive control (calibrator) and serum from a healthy dog as a negative control and all samples were analyzed in duplicate. The result was quantified as ELISA units (EU) related to a positive canine serum used as a calibrator and arbitrarily set at 100 EU. The cutoff was established at 35 U (mean + 4 SD of values from 80 dogs from non- endemic area). Sera were classified as being high positive, when having a positivity percentage (% p) equal or higher than 300% (≥300%), medium positive were classified as % p equal or higher than 150% (>150%) and less than 300% (<300%). Finally, low positive were sera from those dogs with % p lower than 150% (<150%) and higher than 35%.

### Study location and dogs

The subjects involved in the study were two hundred three dogs from Italy, United Kingdom, Cyprus and Spain. All sera samples were collected between 2011 and 2012. All dogs were classified as positive (infected) or negative (not infected) to *L. infantum* by serological and/or molecular diagnostic techniques. Out of 203 dogs studied, 107 were classified as positive (infected) and the rest were classified as negative to infection.

Dogs were allocated into sick or clinically healthy infected groups based on clinical history, a complete physical examination to reveal the presence of clinical signs consistent with the disease, evidence of clinicopathological abnormalities consistent with leishmaniosis and a positive quantitative serology and/or molecular test for *L. infantum* infection [3]. Sera samples were taken for diagnostic purposes and, therefore, ethical approval was not needed.

#### Clinically sick infected dogs

Sera samples from dogs with clinical leishmaniosis (n = 36) came from Cyprus and Italy. Sick dogs from Cyprus (n = 17) were characterized based on presence of clinical signs at physical examination and/or clinicopathologic abnormalities consistent with clinical leishmaniosis [2,3] as well as having a positive result by *Leishmania* real-time PCR [11] in blood and/or conjunctival swabs and a high positive antibody level using two different quantitative ELISA serological tests [8,12]. Sick dogs from Italy (n = 19) came from the San Marco Veterinary Hospital (Padova, Italy). These dogs were diagnosed based on presence of clinical signs at physical examination and/or clinicopathologic abnormalities consistent with clinical leishmaniosis [2,3] based on CBC, serum biochemistry profile, and urianalysis as well as having a positive result by *Leishmania* real-time PCR [9] in blood samples and/or bone marrow and a high positive (n = 15) and medium positive (n = 4) antibody level using UAB in house ELISA [8]. Based on the LeishVet guidelines for staging disease [2,3], all dogs in the group were stage II or above. Therefore, all dogs presented at least moderate disease.

#### Clinically healthy infected dogs

Dogs classified as clinically healthy infected animals were from Cyprus (n = 6) and Spain (n = 12), characterized by the absence of clinical signs based on physical examination and absence of laboratory abnormalities based on CBC and serum biochemistry profile, and with a positive serological result based on UAB in house ELISA [8]. Dogs were classified as high positive (n = 4), medium positive (n = 3) and as low positive (n = 11) based on UAB in house ELISA [8]. In the case of the six dogs from Cyprus, they were also positive with *Leishmania* real-time PCR [11] of blood and/or conjunctival swabs.

#### Seropositive infected dogs with low to high levels of anti-Leishmania antibodies

A total of 53 dogs were studied, no clinical or clinicopathological information was available for these dogs and they were allocated to this group based on anti-*Leishmania* antibody levels detected by UAB in house ELISA [8]. These serum samples were collected from owned dogs in several Italian veterinary clinics and submitted to the San Marco Veterinary Laboratory (Padova, Italy) between 2011 and 2012 in order to establish the serological diagnosis of *L. infantum* infection. Dogs were classified as high positive (n = 34), medium positive (n = 14) and as low positive (n = 5) based on UAB in house ELISA [8].

#### Uninfected dogs from non-endemic areas

Fifty canine sera samples from the Queen Mother Hospital at the Royal Veterinary College (RVC, University of London) were sent to the diagnostic laboratory at RVC for serum biochemical profile or other diagnostic tests such as hormonal assays or serologic tests were included in this study.

All these residual sera samples were negative for *L. infantum* based on UAB in house ELISA [8].

#### Uninfected dogs from endemic area

Thirty two sera samples from clinically healthy dogs from an endemic area (Cyprus), with a negative result for two quantitative in house ELISAs [8,12] and *Leishmania* real-time PCR in blood and/or conjunctival swabs [11] were studied.

#### *Dogs seropositive to other pathogens (to evaluate cross reaction*)

Fourteen samples from the San Marco Veterinary Laboratory with a positive serological IFAT result for different pathogens: *Ehrlichia canis* (n = 5, antibody titers ranging from 1:640 to 1:1280), *Toxoplasma gondii* (n = 1, antibody titer of 1:640), *Rickettsia conorii* (n = 7, antibody titers ranging between 1:640 and 1:1280) and *Anaplasma phagocytophilum* (n = 1, an antibody titer of 1:640) were studied. All these samples were negative for *L. infantum* by the quantitative UAB in house ELISA [8].

### Statistical analysis

Performance measures analysed for each test were: sensitivity, specificity, accuracy, area under curve-receiver operating characteristic (AUC-ROC), Kappa index and the Youden index [10].

The agreement between serological diagnostic techniques and several groups of dogs studied was evaluated by the use of kappa index. The kappa agreement between serological diagnostic techniques was determined as follows: no agreement (k < 0), slight agreement (0 < k <0.2), fair agreement (0.2 < k <0.4), moderate agreement (0.4 < k <0.6), substantial agreement (0.6 < k <0.8) and almost perfect agreement (k >0.8).

In order to better characterize the serological test studied, the Youden index [10] was calculated. The Youden index measures the efficiency of a diagnostic test using a single value, replacing the dual form sensitivity-specificity in such a way that a single index is obtained. This ratio can vary from −1 to 1. If the Youden index is less or equal to 0, the diagnostic test analyzed has no informative value. Thus, a diagnostic test is considered good when the Youden index approaches 1.

Other parameters analyzed were the positive predictive value (PPV) and negative predictive value (NPV). Both parameters are performance measures of the effectiveness of a diagnostic test, dependent on the prevalence of disease in a population. The seroprevalence in dogs living in the Mediterranean basin can range from 5 to 30% depending on the region studied [3]. In this study, the PPV and NPV were calculated taking into account the sensitivity and specificity obtained for each of the serological tests evaluated and with respect to dogs from endemic areas with varying seroprevalence: areas with low seroprevalence (10%) [13] and endemic areas with moderate to high seroprevalence (25%) [14].

For the ROC curve analysis, a confidence interval (CI) (95%) for the area under receiver-operating curve was produced for each test analyzed. A significance statistical level α = 0.05 was used for the confidence interval at 95% (CI) with lower and upper limits acceptable for CI. Swets [15] established three categories to determine the accuracy of a diagnostic technique based on the AUC-ROC. These categories are: high accuracy (0.9 < AUC-ROC ≤ 1), moderate accuracy (0.7 < AUC-ROC ≤ 0.9) and, finally, low accuracy (0.5 < AUC-ROC ≤ 0.7).

The IBM SPSS statistics version 20 program was used. A p value < 0.05 was considered significant.

## Results

### Measures of diagnostic performance

The results of the diagnostic performance measures of the serological tests compared are described in Tables 1 and 2. The sensitivity of the serological tests were as follows; ID Screen^®^ (0.953), *Leishmania* 96^®^ and Leiscan^®^ (0.925), IFAT (0.869) and Speed Leish K^®^ (0.636). Specificity was optimal (1.000) for several tests: ID Screen^®^, Leiscan^®^ and Speed Leish K^®^, followed by IFAT (0.917) and finally the *Leishmania* 96^®^ (0.869).

**Table 1:** Results of measures of diagnostic performance of serological tests studied based on manufacturer’s recommendations and based on ROC cut-off values

**Measures of diagnostic performance**									
	**ID screen**^**®a**^	**ID screen**^**®b**^	**Leiscan**^**®a**^	**Leiscan**^**®b**^	**Leishmania 96**^**®a**^	**Leishmania 96**^**®b**^	**IFAT**^**a**^	**IFAT**^**b**^	**Speed Leish K**^**®**^
**Sensitivity**	0.953	0.963	0.925	0.953	0.925	0.832	0.869	0.813	0.636
**Specificity**	1.000	1.000	1.000	1.000	0.896	0.980	0.917	0.990	1.000
**Accuracy**	0.975	0.980	0.961	0.975	0.911	0.902	0.892	0.897	0.808
**Kappa index**	0.951	0.961	0.921	0.951	0.822	0.804	0.783	0.795	0.622
**Youden index**	0.953	0.963	0.925	0.953	0.821	0.811	0.786	0.803	0.636

^a^Measures of diagnostic performance based on manufacturer’s recommendations.

^b^Measures of diagnostic performance based on ROC cut-off values. The new cut-off established for each test was: ID Screen^®^ (Positive ≥ Ratio 41.97; Negative < Ratio 41.97), Leiscan^®^ (Positive ≥ Ratio 0.77; Negative < Ratio 0.77), Leishmania 96^®^ (Positive ≥ Ratio 0.52; Negative < Ratio 0.52) and IFAT (Positive ≥1:160; Negative <1:160).

**Table 2:** Positive predictive value (PPV) and negative predictive value (NPV) of each serological test based on high (25%) or low seroprevalence settings (10%)

	**ID screen**^®^	**Leiscan**^®^	**Leishmania96**^®^	**IFAT**	**Speed Leish K**^®^
**PVV**					
** High seroprevalence setting (25%)**	1.000	1.000	0.748	0.777	1.000
** Low seroprevalence setting (10%)**	1.000	1.000	0.497	0.538	1.000
**NPV**					
** High seroprevalence setting (25%)**	0.985	0.976	0.973	0.955	0.892
** Low seroprevalence setting (10%)**	0.995	0.992	0.991	0.984	0.961

Accuracy was as follows: ID Screen^®^ (0.975), Leiscan^®^ (0.961), *Leishmania* 96^®^ (0.911), IFAT (0.892) and Speed Leish K^®^ (0.808).

In relation to Kappa agreement for all serological techniques analyzed, there was almost perfect agreement between the ID Screen^®^, Leiscan^®^ and *Leishmania* 96^®^ and dogs with different states of infection (K = 0.951; K = 0.921, K = 0.822; respectively). Substantial agreement was found between IFAT and dogs with different states of infection (K = 0.783) and between the Speed Leish K^®^ and dogs with different states of infection (K = 0.622). Using the Youden index to measure test efficiency, the highest efficiency was obtained by the ID Screen^®^ (0.953), then Leiscan^®^ (0.925), *Leishmania* 96^®^ (0.821), IFAT (0.786) and, finally, the Speed Leish K^®^ (0.636).

In a high seroprevalence setting (25%), the PPV was optimal (1.000) for all tests except *Leishmania* 96^®^ (0.748) and IFAT (0.777). However, in a low seroprevalence setting (10%) different tests performed better for PPV; with the ID Screen^®^, Leiscan^®^ and Speed Leisk K^®^ (1.000), superior to IFAT (0.538) and, finally, the *Leishmania* 96^®^ (0.497).

With relation to the NPV in high seroprevalence areas (25%), negative NPV was optimal for the ID Screen^®^ (0.985), followed by Leiscan^®^ (0.976), *Leishmania* 96^®^ (0.973), and IFAT (0.955) and, finally, the Speed Leish K^®^ (0.892). A similar but not identical profile was seen at low seroprevalence settings (10%) for NPV: ID Screen^®^ (0.995), Leiscan^®^ (0.992), *Leishmania* 96^®^ (0.991), IFAT (0.984) and Speed Leish K^®^ with the lowest value (0.961) (Table 2).

### ROC Curve analysis

**Figure 1:**
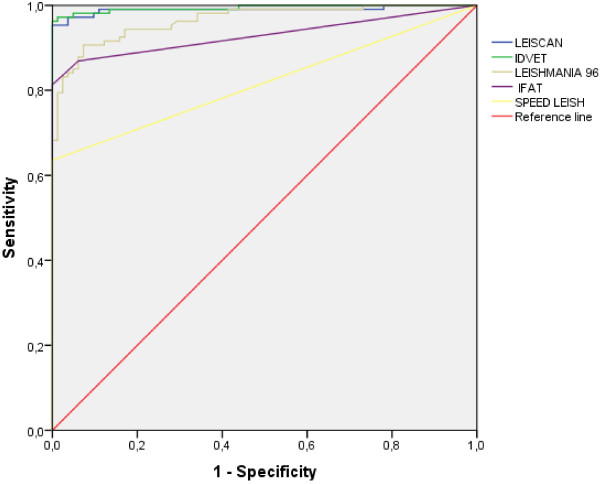
AUC-ROC curve analysis of each serological test studied based on manufacturer’s recommendations.

AUC-ROC analyses and confidence intervals (CI, 95%) obtained from the curve allowed comparison between the different serological tests. The maximum value was reached for the ID Screen^®^ (0.993 95% CI: 0.983 to 1.000), closely followed by Leiscan^®^ (0.990, 95% CI: 0.975 to 1.000), then *Leishmania* 96^®^ (0.962, 95% CI: 0.938 to 0.985), IFAT (0.926, 95% CI: 0.886 to 0.966) and Speed Leish K^®^ (0.818, 95% CI: 0.757 to 0.878). In relation to the classification system proposed by Swets [13], all tests had high accuracy (0.9 < AUC-ROC ≤ 1) except Speed Leish K^®^ that was classified as moderately accurate (0.7 < AUC-ROC ≤ 0.9) (Figure 1).

Statistically significant differences (p < 0.05) were found between the AUC-ROCs of the quantitative serological tests (IFAT, ID Screen^®^, Leiscan^®^ and *Leishmania* 96^®^) and the only qualitative rapid test evaluated (Speed Leish K^®^). There were also statistically significant differences between AUC-ROCs of the ELISAs (ID Screen^®^ and Leiscan^®^) and the IFAT (p < 0.05). However, there was no statistical difference between the AUC-ROCs of *Leishmania* 96^®^ and IFAT (p > 0.05).

Table 1 shows the results of measures of diagnostic performance parameters based on the AUC-ROC curve cut-off values for the quantitative serological test studied.

### Dogs

Table 3 shows the results of measures of diagnostic performance (sensitivity and specificity) for each group studied based on the manufacturer’s recommendations for the serological tests evaluated.

**Table 3:** Results of measures of diagnostic performance (sensitivity and specificity) for each group studied based on the manufacturer’s recommendations of serological tests

**Groups**		**Measures**	**ID screen**^®^	**Leiscan**^®^	***Leishmania *** **96**^®^	**IFAT**	**Speed Leish K**^®^
**Clinically sick infected dogs (n = 36)**		**Sensitivity**	0.972	0.972	0.972	1.000	0.917
**Clinically healthy infected dogs (n = 18)**			0.833	0.667	0.611	0.944	0.167
**Seropositive infected dogs**	**High antibody levels (n = 34)**		1.000	1.000	1.000	0.882	0.853
	**Medium antibody levels (n = 14)**		1.000	1.000	1.000	0.357	0.214
	**Low antibody levels (n = 5)**		0.800	0.800	1.000	1.000	0.000
	**Total (n = 53)**		0.981	0.981	1.000	0.750	0.604
**Uninfected dogs from non-endemic area (n = 50)**		**Specificity**	1.000	1.000	1.000	0.940	1.000
**Uninfected dogs from endemic areas (n = 32)**			1.000	1.000	0.875	0.938	1.000
**Dogs seropositive to other pathogens (n = 14)**			1.000	1.000	0.571	0.786	1.000

#### Clinically sick infected dogs (n = 36)

The IFAT was positive for all dogs in the group. The commercial tests analyzed obtained a negative result in some animals: Speed Leish K^®^ (3/36), Leiscan^®^ (1/36), ID Screen^®^ (1/36) and *Leishmania* 96^®^ (1/36). One dog was classified as negative by all commercial serological tests while found as high positive by the UAB in house ELISA and positive by IFAT (antibody titer of 1:40). This dog was classified as stage IV based on LeishVet guidelines [2,3] and was confirmed by positive blood and bone marrow real time PCR [9]. Speed Leish K^®^ conflicting results (n = 3) were classified as medium positive (n = 1) and high positive (n = 2) by the UAB in house ELISA.

#### Clinically healthy infected dogs (n = 18)

A higher number of dogs in this group had negative results depending on the serological test evaluated: IFAT (1/18), ID Screen^®^ (3/18), Leiscan^®^ (6/18), *Leishmania* 96^®^ (7/18) and Speed Leish K^®^ (15/18). With respect to the quantitative tests evaluated, all the samples with conflicting serological results were classified as low positive by the UAB in house ELISA, differently from the Speed Leish K^®^ conflicting sample results (n = 15) which were classified as low positive (n = 11), medium positive (n = 1) and high positive (n = 3) by the UAB in house ELISA.

#### Seropositive infected dogs with low to high levels of anti-Leishmania antibodies (n = 53)

All dogs in this group were positive using the *Leishmania* 96^®^. However, some of the dogs in this group were negative by the Leiscan^®^ and ID Screen^®^ (1/53), IFAT (13/53) and the rapid test Speed Leish K^®^ (21/53). Samples with Leiscan^®^ and ID Screen^®^ conflicting results (n = 1) were classified as low positive by the UAB in house ELISA. IFAT results (n = 13) were classified as medium positive (n = 9) and high positive (n = 4) and, finally, Speed Leish K^®^ results (n = 21) were classified as low positive (n = 5), medium positive (n = 11) and high positive (n = 5) by the UAB in house ELISA.

#### Uninfected dogs from non-endemic area (n = 50)

Three dogs were positive based on IFAT with an antibody titer of 1:40. All dogs were seronegative for the other serological tests studied.

#### Uninfected dogs from endemic areas (n = 32)

Two dogs were positive based on IFAT (titer 1:40), whereas for the *Leishmania* 96^®^ ELISA test, 4 dogs had a positive result (4/32). All dogs were negative for the other serological tests studied.

#### Dogs seropositive to other pathogens (n = 14)

The ID screen^®^, Leiscan^®^ and Speed Leish K^®^ where highly specific and no dogs within this group had a positive result with these tests. For the IFAT test, 3 dogs were positive for *L. infantum* antigen (antibody titers of 1:40 and 1:80) with sera positive against different pathogens: *A. phagocytophilum* (antibody titer of 1:640), *E. canis* (antibody titer of 1:1280) and *R. conorii* (antibody titer of 1:1280).

The test with the highest positive results was *Leishmania* 96^®^ (n = 6). Sera positive for *E. canis* (n = 2 with titers ranging from 1:640–1:1280), *R. conorii* (n = 3 with titers ranged from 1:640–1:1280) and *T. gondii* (n = 1 with an antibody titer of 1:640) were all positive for *L. infantum* antigen with this test. However, all positive results were low.

### Using Speed Leish K^®^ as a screening test for CaniLeish^®^ prevaccination

In the present study, some canine sera that were negative by the Speed Leish K^®^, were positive based on quantitative serological techniques. 31 dogs were positive with the Leiscan^®^ and 41 were positive by the *Leishmania* 96^®^ but negative with the Speed Leish K^®^. With ID Screen^®^, 34 positive dogs were found to be negative using the Speed Leish K^®^. For IFAT, 38 positive dogs were negative using the Speed Leish K^®^. Thirty six percent of infected dogs (39/107) were considered negative by this test. Of the 107 infected dogs, a total of 18 (16.82%) with a positive result by all quantitative serological tests were classified as seronegative based on the Speed Leish K^®^. These dogs had false negative results by this rapid test. Table 4 describes number of seropositive animals and antibody levels based on the UAB in house ELISA and IFAT of dogs classified as seronegative by the Speed Leish K^®^.

**Table 4:** **Number of seropositive dogs and antibody levels (UAB in house ELISA and IFAT) of those classified as seronegative by the Speed Leish K**^®^

**UAB in house ELISA**	**Low positive**	**Medium positive**	**High positive**		
**N° dogs**					
**(% positivity; Mean ± SD)**	16 (87.86 ± 32.23) EU	13 (228.21 ± 50.93) EU	10 (327.51 ± 36.93) EU		
**ELISA Unit (EU)**					
**Titers IFAT**	**1:40**	**1:80**	**1:160**	**1:320**	**1:2560**
**N° dogs**	6	8	13	1	2

## Discussion

Serological methods such as IFAT, ELISA and rapid tests are amongst the most common diagnostic techniques employed in clinical and research studies on canine *L. infantum* infection [3,16]. For both IFAT and ELISA, quantification using antibody titer or optical density allows classification of antibody levels against *L. infantum* antigen. The IFAT technique has traditionally been considered a gold standard for the serological diagnosis of *L. infantum* infection, with optimal performance measures with regards to sensitivity and specificity [14]. This test is still considered by some authors to be a technical reference in diagnostic laboratory practices [17]. However, its interpretation can be subjective depending on the operator’s skills and experience when interpreting results [15]. The ELISA technique allows use of different kinds of antigens. These antigens can be classified into four groups according to their nature: whole or soluble extracts of promastigotes, whole or soluble extracts of amastigotes, recombinant proteins and purified proteins. The sensitivity and specificity of the ELISA technique varies depending on which antigen is used [16,18]. The use of amastigotes as antigen appears to be more sensitive than promastigote antigen for detection of antibodies in both sick and subclinical dogs [19]. In the present study, the serological techniques with better diagnostic performance measures were found to be the quantitative ELISAs. The performance measures for ELISA sensitivity ranged from 0.925 to 0.953 and specificity ranged from 0.869 to 1.000. The IFAT technique obtained a sensitivity of 0.869 and a specificity of 0.917. These results are similar to those in other published studies [18,20]. It is noteworthy that, apart from the UAB in house ELISA test, IFAT was the only one test able to detect all the subjects belonging to the group of clinically sick infected dogs (36/36) and almost all the ones belonging to the group of clinically healthy infected dogs (17/18). The worst performances of IFAT were observed in the group of seropositive infected dogs with medium to high levels of anti-*Leishmania* antibodies (13/53 false negative results) but it is important to note that this group of dogs were classified based only on a quantitative UAB in house ELISA precluding a possible certain diagnostic performance bias although not likely due to the fact the high antibodies levels are associated with parasite dissemination and clinical illness [1]. Another limitation of IFAT (at the cut-off of 1:40) was an imperfect specificity observed in the group of uninfected dogs from non-endemic area (3/50) but also in dogs seropositive to other pathogens (2/32) possibly due to cross-reactions with other pathogens such as *A. phagocytophilum*, *E. canis* and *R. conorii*.

The use of ROC curve analysis as an analytical tool in comparative studies of diagnostic tests is quite widespread, however, its use in veterinary studies has been less common than in human research. The main advantage of this type of analysis is to select the most optimal cut-off [21]. In this study, the ROC curve analysis managed to improve the performance measures of ELISAs: ID Screen^®^ and Leiscan^®^. A new cut-off established for Leishmania 96^®^ and the in house IFAT produced an increase in specificity; however, this was at the expense of reduced sensitivity. Finally, the ROC curve cannot provide a cut-off that will maximize performance measures for the rapid Speed Leish K^®^ test, in which the result is a dichotomous variable.

Immunochromatographic rapid tests such as Speed Leish K^®^ have many advantages: they are easy to interpret, quick to use and do not require sophisticated equipment and so may be ideal for use in clinical practice. However, the rapid test only provides a qualitative result and the majority of the times requires confirmation with a quantitative serological test which may increase the cost of diagnosis. Diagnosis using a quantitative serological technique is always advisable as it provides more information regarding antibody level [2,3]. The Speed Leish K^®^ showed high specificity with a value of 1.000 in this study, however, sensitivity was low (0.636). A previous study has shown that qualitative rapid tests have low sensitivity in infected subclinical dogs [18] and this was also demonstrated in this study.

A study published by Ferroglio *et al*. [6] evaluated a total of 250 samples, of which 125 were negative and 125 were positive to *L. infantum* infection determined by IFAT. Of the 125 positive samples, 81 samples were strongly positive (antibody titers ≥ 1: 160) and 44 samples showed low-reactivity (antibody titers of 1:40 or 1:80). Samples with a titer of 1:40 and 1:80 by IFAT were evaluated again with Western Blot (WB) because it is considered a more sensitive technique than IFAT [22]. In this study, the sensitivity and specificity for Speed Leish K^®^ were 0.963 and 1.000 based on strongly positive and negative samples by IFAT. In the case of samples with borderline results for IFAT (antibody titers of 1:40 or 1:80), when WB was considered the reference technique, Speed Leish K ^®^ showed a sensitivity and specificity of 0.975 and 1.000, respectively for sera with antibody titers of 1:80. For sera with IFAT titers of 1:40, the sensitivity of Speed Leish K ^®^ was 0.533 and the specificity was 1.000. This study has found that dogs with a low level of anti-*Leishmania* antibodies may not be detected correctly by the rapid test Speed Leish K^®^[6] in agreement with the present results of this study. In the present study, Speed Leish K^®^ detected only 3 dogs out of 18 seropositive infected healthy dog group. This study has also shown that Speed Leish K^®^ failed to detect infected dogs (39/107). In addition, a total of 18 out of 107 infected dogs (16.82%) with a positive result by all quantitative serological tests were classified as seronegative based on Speed Leish K^®^. Moreover, Speed Leish K^®^ did not detect three sick infected dogs. It is important to highlight that a screening serological test should have a good sensitivity. Therefore, the Speed Leish K^®^ does not seem to have a good diagnostic performance as a screening serological test.

The Speed Leish K^®^ has been recommended for prevaccination screening for *L. infantum* infection before use of the CaniLeish^®^ vaccine. The manufacturers recommend vaccination of those dogs with a negative Speed Leish K^®^ result. The efficacy of the vaccine has been evaluated exclusively on negative *Leishmania* dogs and therefore its use is limited to non-infected seronegative healthy dogs [23]. The results of this study demonstrate that the Speed Leish K^®^ test has low sensitivity. The use of this test may result in vaccinating dogs that are infected with *L. infantum* and seropositive or even sick but that appear seronegative based on the rapid test Speed Leish K^®^ as demonstrated in the present study. The consequences of immunizing seropositive dogs are unknown, but there is a risk that some vaccinated seropositive dogs could develop clinical leishmaniosis. In addition, the incorrect diagnosis of infected dogs may have important implications for Veterinary Medicine and Public Health that should be considered. Future studies should further characterize the efficacy and possible implications of vaccination of seropositive dogs.

A study [20] evaluated the Leiscan^®^ test and obtained good performance measures of sensitivity and specificity of 0.980 and 1, respectively, similar to those described in the present study (sensitivity of 0.925 and specificity 1). The main difference between the two studies is the type of sample used. The dogs studied in the cited article were experimentally infected intravenously with a high dose of promastigotes [20], whilst the present study enrolled uninfected and naturally infected dogs. The evolution and pathogenesis of natural infection is highly variable and not easily comparable with experimental infection. It should be pointed out that the administration of intravenous parasites for experimental infection induces a high production of anti-*Leishmania* antibody levels, and a rapid progression of clinical signs and lesions when compared to experimental intradermal infection [24]. It is known that naturally sick infected dogs have higher production of antibodies directed to a greater number of antigenic epitopes [25,26] and these antibodies are more easily detectable by a serological test, compared to subclinical infected dogs in which the production of antibodies and the number of antigenic epitopes to which the immune system responds is lower. For this reason, it is important to note that there may also be differences between experimental and natural infections in the degree and type of antibody production.

The *Leishmania* 96^®^ has also been evaluated in other studies [27]. Both sensitivity (88.9% *versus* 92.5% of this study) and specificity (78.2% *versus* at 89.6% in the present study) are mildly different with better diagnostic performance in the present study. A possible explanation for the difference in both sensitivity and specificity between the two studies could be the use of a single serological method as a reference test and poor characterization of dogs in the previous study. The good sensitivity and specificity of ID Screen^®^ in the present study are very similar to those found in a previous study [28].

One of the major disadvantages of serological tests is the possibility of cross-reaction phenomenon against other pathogens or other clinical entities [19,29]. A total of 14 samples from animals diagnosed by the IFAT technique with a positive result to other pathogens were selected for this study to determine specificity. The only serological tests that had a specificity of 100% were the Leiscan^®^, ID Screen^®^ and Speed Leish K^®^, whilst other tests studied showed some cross-reactions with *E. canis*, *A. phagocytophilum*, *R. conorii* and *T. gondii*. This cross-reactivity phenomenon against other pathogens such as *E. canis*, *Babesia canis*, *T. gondii*, *Neospora caninum* and *Hepatozoon canis* has been sporadically described in other studies [19,29,30]. Cross-reactivity with *L. infantum* is more common in infection of dogs with other species of *Leishmania* or with other protozoans such as *Trypanosoma cruzi,* which are prevalent in America and not in Europe [31,32]. Cross-reactions typically result in false positive results with low antibody levels [19] in agreement with the results of the present study.

The absence of a diagnostic reference or gold standard results in the combination of one [27], two [33] or more diagnostic tests [29] used as “gold standard” in several studies from which new techniques can be compared. A problem associated with this lack of “gold standard” is that a standardization of results is not possible, which means that comparison of results between studies is difficult. In addition, there is a clear tendency in many studies to select only sick dogs with clinical signs and there are limited studies with descriptions of clinical staging and severity of illness, thus, an imbalance between studies on infected sick dogs and subclinical infected dogs exist [19,34]. This type of approach can lead to obtaining higher values of diagnostic performance measures than would be achieved with more heterogenous dog groups. In the present study, we used the new clinical classification previously described [2,3] and, in addition, all sick dogs presented at least moderate disease based on the LeishVet clinical staging [3]. Moreover, we studied different states of infection of dogs. In future comparative studies of diagnostic and serological tests, it would be advisable to include other independent diagnostic tests as a reference; namely quantitative real-time PCR (RT-PCR) or assessments of the cellular immunity test as we did in some groups in the present study.

## Conclusions

This study demonstrated that all serological techniques showed high specificity. However, sensitivity varied from one technique to another. The Leiscan^®^ and ID Screen^®^ tests had superior diagnostic performance measures compared to the IFAT, but among the tests evaluated in the present study, IFAT was the most sensitive test for the confirmation of clinically sick infected dogs. All the quantitative serological tests were superior when compared to the only qualitative rapid test evaluated (Speed Leish K^®^). The use of Speed Leish K^®^ as a screening test prior to the use of the vaccine CaniLeish^®^ may not be appropriate as sensitivity was found to be substantially lower than for the quantitative ELISAs and IFAT. This may lead to the vaccination of seropositive dogs and in some cases seropositive sick dogs.

## Abbreviations

AUC-ROC: Area under curve-receiver operating characteristics; CanL: Canine leishmaniosis; CBC: Complete blood count; CI: Confidence intervals; DNA: Deoxyribonucleic acid; ELISA: Enzyme-linked immuno sorbent assay; EU: ELISA unit; IFAT: Indirect fluorescent antibody test; IgG: Immunoglobulin G; NPV: Negative predictive value; PCR: Polymerase chain reaction; PPV: Positive predictive value; RVC: Royal Veterinary College; UAB: Universitat Autònoma de Barcelona.

## Competing interests

The authors declare that they have no competing interests.

## Authors’ contributions

LSG designed the study, set up UAB in house ELISA, contributed with data analysis and interpretation and wrote the manuscript. SV performed ELISAs testing and rapid test, contributed with data analysis and interpretations and wrote the manuscript. MC performed ELISAs testing and rapid test and contributed with data analysis. MT collected and provided some of canine sera samples, and performed DNA extraction, and real-time PCR. TF provided some of canine sera samples and contributed with interpretation of results. AN supervised execution of IFAT test and contributed with interpretation of results. All authors read and approved the final manuscript.
